# Xuanfei Pingchuan Capsules Ameliorate Autophagy in Human Bronchial Epithelial Cells by Inhibiting p38 Phosphorylation

**DOI:** 10.3389/fphar.2021.748234

**Published:** 2021-12-02

**Authors:** Xiaoming Xue, Lihong Meng, Hongyu Cai, Yaoqin Sun, Ye Zhang, Hao Li, Yu Kang, Bobo Zhou, Fang Shang, Wei Guan, Li Zhang, Xu Chen, Luodan Zhang

**Affiliations:** Department of Respiration, Shanxi Traditional Chinese Medicine Hospital, Taiyuan, China

**Keywords:** chronic obstructive pulmonary disease, Xuanfei Pingchuan capsules, cigarette smoke extract, human bronchial epithelial cells, p38 phosphorylation, autophagy

## Abstract

**Background:** This study aimed to investigate the protective effect of Xuanfei Pingchuan Capsules (XFPC) on autophagy and p38 phosphorylation in human bronchial epithelial (HBE) cells induced by cigarette smoke extract (CSE).

**Methods:** HBE cells were divided into five groups: blank, CSE, low XFPC dose (XFPC-L), medium XFPC dose (XFPC-M), and high XFPC dose (XFPC-H). HBE cells were induced by CSE to establish a cell model for chronic obstructive pulmonary disease, and different doses of XFPC medicated serum were used to treat the cells. The Cell Counting Kit-8 was used to detect cell viability. Flow cytometry was used to detect cell apoptosis. Fluorescence microscopy and the expression level of microtubule-associated protein light chain 3 (LC3)-II in immunohistochemical method were used to observe autophagy in cells. Western blot was used to detect the protein expression level of p38, phospho-p38 (p-p38), LC3-I, LC3-II and Beclin 1. Real-time polymerase chain reaction was used to detect the expression of *LC3-I*, *LC3-II* and *Beclin 1* on mRNA level.

**Results:** Compared with the blank group, the cell viability of the CSE group was significantly decreased, and apoptosis and the level of autophagy in cells were significantly increased. The mRNA and protein expression of LC3-I, LC3-II, Beclin 1 and the protein level of p-p38 were significantly increased in the CSE-HBE cells. Compared to the CSE group, the different doses of XFPC medicated serum increased cell viability, decreased cell apoptosis, and inhibited mRNA and protein expression of LC3-I, LC3-II, Beclin 1 and protein level of p-p38. These results were especially observed in the group XFPC-H. After adding a p38 agonist, the therapeutic effect of XFPC on cell viability and autophagy was suppressed.

**Conclusion:** XFPC significantly increased cell viability in a CSE-induced HBE cell model for chronic obstructive pulmonary disease through inhibiting the level of autophagy mediated by phosphorylation of p38.

## Introduction

Chronic obstructive pulmonary disease (COPD) is a major clinical inflammatory lung disease caused by smoking and alveolar abnormalities. COPD has high prevalence, mortality, and disability rates and a heavy burden of disease (Singh et al., 2019). Smoking is the primary risk factor for COPD, followed by air pollution, exposure to solid fuel smoke, occupational exposure, and *Pseudomonas aeruginosa* infection (Chan et al., 2019; De Matteis et al., 2019; Doiron et al., 2019; Eklof et al., 2020). The standard treatment for COPD is inhaling glucocorticoids and bronchodilators; however, these drugs can cause adverse reactions, such as aggravating infection and inducing osteoporosis and cataracts (Agusti Alvar et al., 2018). Adverse events such as these have a heavy economic burden for patients. Therefore, it is necessary to seek a new method of treatment for COPD.

The molecular mechanism of COPD pathogenesis remains unclear (Wang et al., 2019). Many studies have shown that abnormal activation of autophagy is a common feature of the lung epithelial cells of COPD patients, mouse models, and cell culture model systems (Vij et al., 2018; Bodas et al., 2019). Additionally, autophagy dysfunction caused by cigarette smoke has been shown to exacerbate lung aging and COPD-induced emphysema (Racanelli et al., 2018; Vij et al., 2018). These studies indicate that autophagy is closely related to the pathogenesis of COPD, potentially via airway remodeling and lung parenchyma changes. Thus, autophagy dysfunction provides a new target for the clinical treatment of COPD.

Autophagy is a process of automatic cellular component degradation that controls protein and organelle lysosomal degradation (Hikichi et al., 2019). An increasing number of studies have found that autophagy plays an important role in maintaining homeostasis of the intracellular environment and protecting cells from various damages (Liao et al., 2019). Thus, many signal pathways and macromolecular signal complexes are involved in autophagy. However, when autophagy is dysregulated by smoking, environmental pollution, aging, and other factors, this cellular process may become overburdened or imbalanced, leading to cell invasion, reactive oxygen species production, and, ultimately, the development of COPD (Tan et al., 2019). Autophagy involves dozens of related proteins. Beclin 1 (a homolog of yeast atg6) and (microtubule associated protein one light chain 3) are crucial. Beclin 1 can regulate the shape of autophagy precursors. LC3 participates in the formation of autophagosome membranes. Under normal circumstances, LC3 exists in the cytoplasm in an unlipidized, soluble form, called LC3-I. When autophagy is formed, LC3-I is enzymatically converted into membrane-type LC3, also known as LC3-II. LC3-II remains on the autophagosome membrane stably until it fuses with the lysosome, so it is always used as a marker for autophagosomes. From the start of autophagosome production to autophagolysosome degradation, the content of LC3-I and LC3-II is constantly changing during the whole process. However, in mammalian cells, the total level of LC3 is generally constant, so the autophagy activity can be reflected by the content and ratio of LC3-I and LC3-II. When the autophagy level of COPD patients increases, the expression of LC3-I decreases but that of LC3-II increases.

From the point of view of Traditional Chinese Medicine, the pathological nature of COPD is asthenia in both origin and superficiality. Xuanfei Pingchuan Capsules (XFPC) are a proprietary Chinese medicine preparation of Shanxi Traditional Chinese Medicine Hospital. XFPC have a significant effect on COPD, especially in patients with Intermingled Phlegm and Blood Stasis Syndrome obstructing the lungs. Previous clinical and experimental studies have confirmed the curative effect of XFPC on COPD (Xiaoming et al., 2012; Xiaoming et al., 2017). Therefore, this study aimed to investigate the effect of XFPC on cigarette smoke extract (CSE)-induced autophagy in human bronchial epithelial (HBE) cells.

## Methods

### Drugs and Reagents

The ingredients of XFPC include *roasted ephedra* (10 g), *almonds* (10 g), *Scutellaria* (10 g), *perillaseed* (10 g), *Platycodon grandiflorum* (10 g), *cortex mori radices* (10 g), *Aster* (15 g), *Flos Farfaraes* (15 g), *lepidium seed* (10 g), *Pinellia ternata* (10 g), *liquorice* (licorice, 10 g), *Schisandra* (10 g), *Ginkgo biloba* (10 g), *Angelica* (15 g), *Astragalus* (30 g), and one pair of *Gecko*. XFPC were provided by the preparation room of Shanxi Academy of Traditional Chinese Medicine (Taiyuan, China).

Anisomycin was purchased from Selleck Chemicals (Houston, TX, United States). Primary antibodies against LC3B, phospho-extracellular signal-regulated kinase, phospho-p38 (p-p38), β-actin, and horseradish peroxidase-conjugated secondary antibodies were all purchased from Cell Signaling Technology (Danvers, MA, United States). Flow Cytometer Kit was purchased from Cell Signaling Technology (Danvers, MA, United States). The SuperSignal West Pico Chemiluminescent Substrate for horseradish peroxidase enzyme was obtained from Soleibao Technology Co., Ltd. (Beijing, China). Cell Counting Kit-8 (CCK8) was purchased from Soleibao Technology Co., Ltd. (Beijing, China).

### Preparation of XFPC Medicated Serum

Twelve healthy Sprague-Dawley rats were randomly divided into four groups: blank, low XFPC dose (XFPC-L), medium XFPC dose (XFPC-M), and high XFPC dose (XFPC-H). The blank group was given intragastric saline. The XFPC groups were given different doses of XFPC solution twice daily for 5 days: XFPC-L group, 2.7 g/kg; XFPC-M group, 5.4 g/kg; and XFPC-H group, 10.8 g/kg. One hour after the last gavage, the animals were anesthetized with ether and blood was taken from the abdominal aorta under aseptic conditions. The serum was inactivated in a water bath at 56°C for 30 min, filtered through a microporous membrane, and stored at −20°C.

### Preparation of CSE

Mainstream smoke from 10 cigarettes (1.1 mg nicotine and 11 mg tar per cigarette; Hongta Group, Yuxi, China) was slowly drawn into a 50-ml syringe and bubbled through 50 ml of Dulbecco’s Modified Eagle Medium that had been pre-warmed in a water bath at 37°C. The preparation, considered to be 100% CSE, was titrated to pH 7.4, sterilized with a 0.22 μm filter (Millipore, Bedford, MA, United States), and stored at −80°C. Serum-free cell culture medium was used to dilute the 100% CSE solution to the required CSE concentrations.

### Cell Culture and Model

HBE cells obtained from Lixiao Biomedical Technology Co., Ltd. (Hangzhou, China) were cultured at 37°C in 5% CO_2_-enriched air in Dulbecco’s Modified Eagle Medium supplemented with 10% fetal bovine serum, 50 U/mL penicillin G sodium, and 50 μg/ml streptomycin sulfate. All experiments were performed on logarithmically growing cells. Cell layers were 70–80% confluent at the time of CSE exposure. The HBE cells were induced by CSE for 24 h to construct a cell model for COPD.

### Flow Cytometry

For all cell model for COPD, different doses of XFPC medicated serum and CSE-HBE cells were incubated together for 5 h the cells were collected, washed three times with PBS, and resuspended in cold 500 µl 1 × binding buffer, mixed with 5 µl of Annexin-V-fluorescein isothiocyanate (FITC) and 2.5 µl of propidium iodide (PI), and eventually detected using a FACSAria Sorter.

### Cell Viability Assay

Cells were seeded at an initial density of 5,000 cells per well onto 96-well plates and allowed to attach overnight. Cells were then treated with the indicated concentrations of CSE, XFPC, or a combination. Cells had been pretreated with different doses of XFPC medicated serum 1 h before CSE exposure. After 24 h, 10 μL of reagent from the Cell Counting Kit-8 (CCK8, Soleibao Technology Co., Ltd., Beijing, China) was added to each well and the plates were incubated for 2 h. Absorbance values were recorded using the Model 680 Microplate Reader (Bio-Rad, Hercules, CA, United States) at an absorbance of 450 nm.

### Immunohistochemistry

After 24 h of CSE treatment, the different concentrations of XFPC medicated serum and CSE-HBE cells were incubated together for 5 h. The cells were then washed with phosphate-buffered saline (PBS) three times, fixed with 4% paraformaldehyde for 15 min, and again washed three times with PBS. The cells were incubated with 0.5% TritonX-100 for 20 min, washed with PBS three times, and shaken. The cells were blocked with 5% bovine serum albumin (BSA) for 60 min. The primary antibody was added (LC3B) (Abcam Trading Co., Ltd., Shanghai, China), the cells were incubated at 4°C overnight with shaking, and then washed with tris-buffered saline (TBS) with 0.1% Tween. The secondary antibody was added and the cells were incubated overnight. A fluorescence microscope was used to visualize the cells.

### Western Blot Analysis

Cells were lysed in radioimmunoprecipitation assay buffer containing 50 mM Tris–HCl (pH 7.4), 150 mM NaCl, 1% NP-40 lysis buffer, 0.5% sodium deoxycholate, 2 mM sodium fluoride, 2 mM ethylenediaminetetraacetic acid, 0.1% sodium dodecyl sulfate (SDS), and phenylmethylsulfonyl fluoride. Protein concentrations were determined using a bicinchoninic acid protein assay kit (Seth Biological Technology Co., Ltd., Xi’an, China). Total protein (20 μg) was fractionated using 10% SDS polyacrylamide gel electrophoresis and transferred to polyvinylidene difluoride membranes. Membranes were blocked for 1 h at room temperature with 5% BSA in TBS-Tween and incubated overnight at 4°C with the appropriate primary antibodies (LC3-I, LC3-II, Beclin 1, p38 and p-p38). After incubation with horseradish peroxidase-conjugated second antibodies, the immune complexes were detected using the SuperSignalWest Pico Chemiluminescent Substrate (Soleibao Technology). Band intensities were quantified using computerized image analysis.

### Real-Time Polymerase Chain Reaction

The total RNA in cells was extracted using the TRIzol reagent (ThermoFisher Scientific, Waltham, MA, United States) and reverse transcribed to cDNA using the PrimeScript RT Reagent Kit (Takara Bio Inc., Shiga, Japan) according to the manufacturer’s instructions. Real time-polymerase chain reaction (RT-PCR) was performed on the cDNA samples using the SYBR Premix Ex Taq II master mix (TAKARA, Wuhan, China). The reaction conditions were as follows: 95°C for 30 s, followed by 40 cycles of denaturation at 95°C for 5 s, annealing at 60°C for 30 s, and extension at 60°C for 34 s. All amplifications were performed in triplicate. Information of RT-PCR primers are shown in the Table 1.

**TABLE 1:** Primer information for real-time polymerase chain reaction.

Gene name	Primer Sequence (5′ to 3′)	Post-primer (5′ to 3′)
LC3I	GAG​GAT​CCA​TGC​CCT​CCG​ACC​GGC​CTT​TC	CAG​AAG​CTT​CAG​AAG​CCG​AAG​GTT​TCT​TG
LC3II	CCA​GGA​AAC​CTT​CGG​CTT​CTG​A	TCA​GAA​GCC​GAA​GGT​TTC​CTG​G
Beclin-1	TAG​GAT​CCA​TGG​AAG​GGT​CTA​AGA​C	GCG​AAG​CTT​TCA​TTT​GTT​ATA​AAA​T-
GAPDH	AAC​GGA​TTT​GGT​CGT​ATT​GGG	TGG​AAG​ATG​GTG​ATG​GGA​TTT​C

Data were analyzed using the QuantStudio™ seven Flex RT-PCR system (ThermoFisher). Cycle threshold (CT) values were analyzed using the comparative CT (ΔΔCT) method. The relative amount of target mRNA (2^−ΔΔCT^) was obtained by normalizing the values to endogenous glyceraldehyde 3-phosphate dehydrogenase (GAPDH).

### Statistical Analysis

All data are expressed as mean ± standard deviation and analyzed using SPSS version 20.0 (IBM Corp., Armonk, NY, United States). One-way analysis of variance (ANOVA) followed by Tukey’s HSD test were used to analyze normally-distributed data. Lowercase letters indicate statistically significant differences (*p*-value < 0.05).

## Results

### XFPC Improved the Cell Survival Rate and Inhibited Apoptosis in the CSE-HBE Cell Model

**FIGURE 1:**
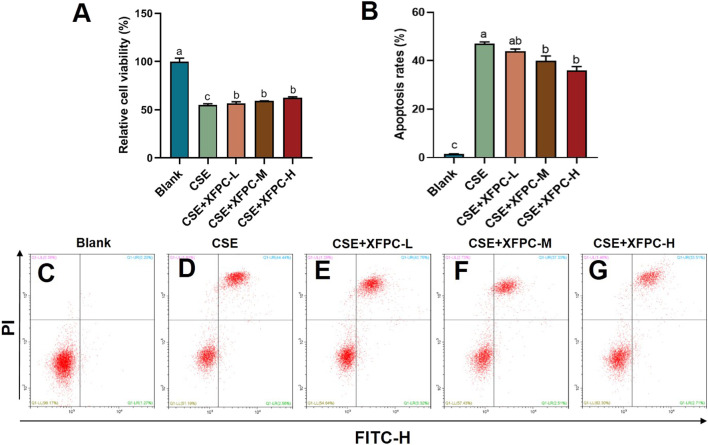
XFPC could improve the cell survival rate and inhibit the apoptosis in CSE-HBE cells model. **(A)** Effects of different concentrations of XFPC on cell viability in CSE-exposed HBE cells. **(B)** The apoptosis rates of HBE cells in different group in panels **(C–G)**. **(C–G)** CSE-exposed HBE cells were treated with different concentrations of XFPC or a blank group. Apoptosis was analyzed by flow cytometry after Annexin V/PI double staining. Values are presented as mean ± SD. Lowercase letters indicate statistically significant differences (*p* < 0.05) according to ANOVA followed by Tukey’s HSD test. XFPC, Xuanfei Pingchuan Capsules; CSE, cigarette smoke extract; HBE cells, human bronchial epithelial cells.

We used the CSE-induced HBE cells to construct a cell model for COPD. Then different concentrations of XFPC medicated serum were used to treat cells. We found that CSE exposure significantly inhibited cell viability, and XFPC could increase the cell viability of CSE-HBE in a dose-dependent manner (Figure 1A). Otherwise, CSE exposure significantly promoted HBE cell apoptosis to 40% compared with the blank group (Figures 1B–D). After the intervention of different concentrations of XFPC medicated serum, the apoptosis rate was significantly reduced, especially in the XFPC-M and XFPC-H groups (Figures 1B,E–G).

### XFPC Significantly Reduced Autophagy Levels in the CSE-HBE Cell Model

**FIGURE 2:**
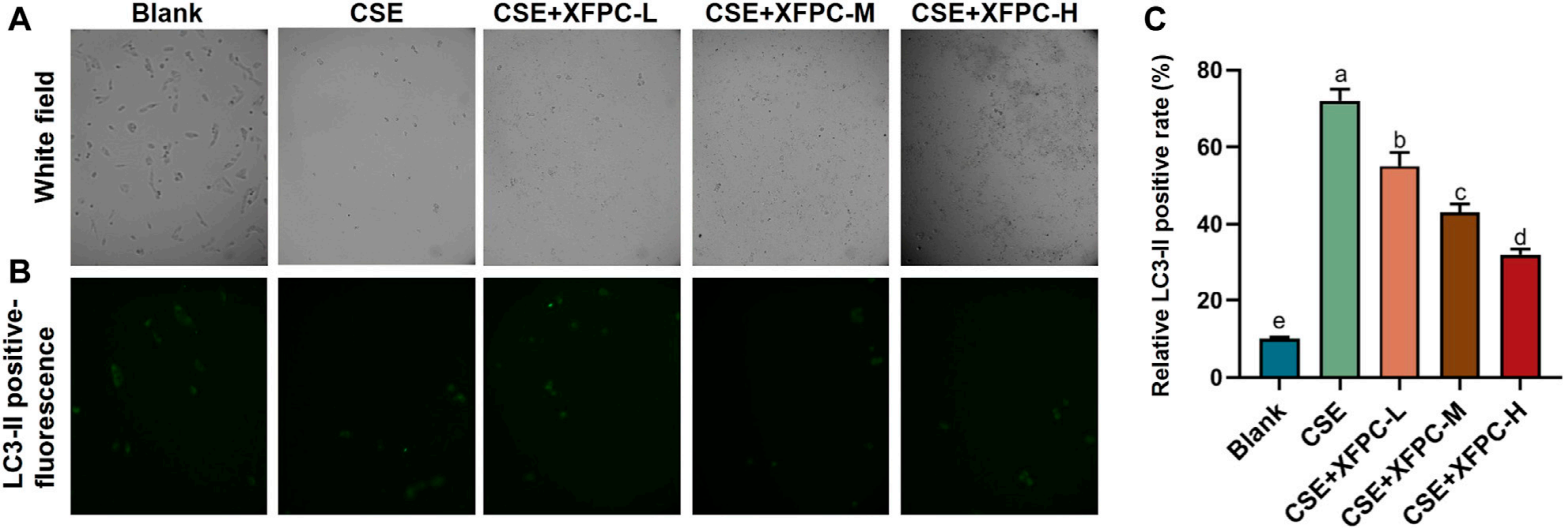
XFPC significantly reduces the autophagy levels of CSE-HBE cell model. **(A)** The cell morphology of HBE cells treated with CSE and different concentrations of XFPC. **(B)** The formation of LC3-II puncta in each group corresponding with panel **(A)** were analysed by immunofluorescence under microscopy (×200). **(C)** The relative LC3-II positive rate indicating level of autophagy was shown. Lowercase letters indicate statistically significant differences (*p* < 0.05) according to ANOVA followed by Tukey’s HSD test. XFPC, Xuanfei Pingchuan Capsules; CSE, cigarette smoke extract; HBE cells, human bronchial epithelial cells.

Compared to the blank group, the number of cells in the COPD model was drastically reduced, the morphology was severely damaged, and cells contorted into a round shape. Different doses of XFPC were used to treat the cells in the model. After treatment, the HBE cells showed an elongated shape after attaching to the wall, and the number of cells increased (Figure 2A). The immunofluorescence test of LC3-II protein showed that CSE activated autophagy in HBE cells, leading to the accumulation of autophagosomes. XFPC reduced the level of autophagy in HBE cells, with the degree of inhibition being positively correlated with the dose of XFPC (Figure 2B). Moreover, the number counted in each group also suggested that the indicator of autophagy LC3-II-GFP positive cells were induced by CSE treated and rescued partially by XFPC cured (Figure 2C).

### XFPC Inhibited Expression of Autophagy Indicator Proteins in CSE-HBE Cell Model

**FIGURE 3:**
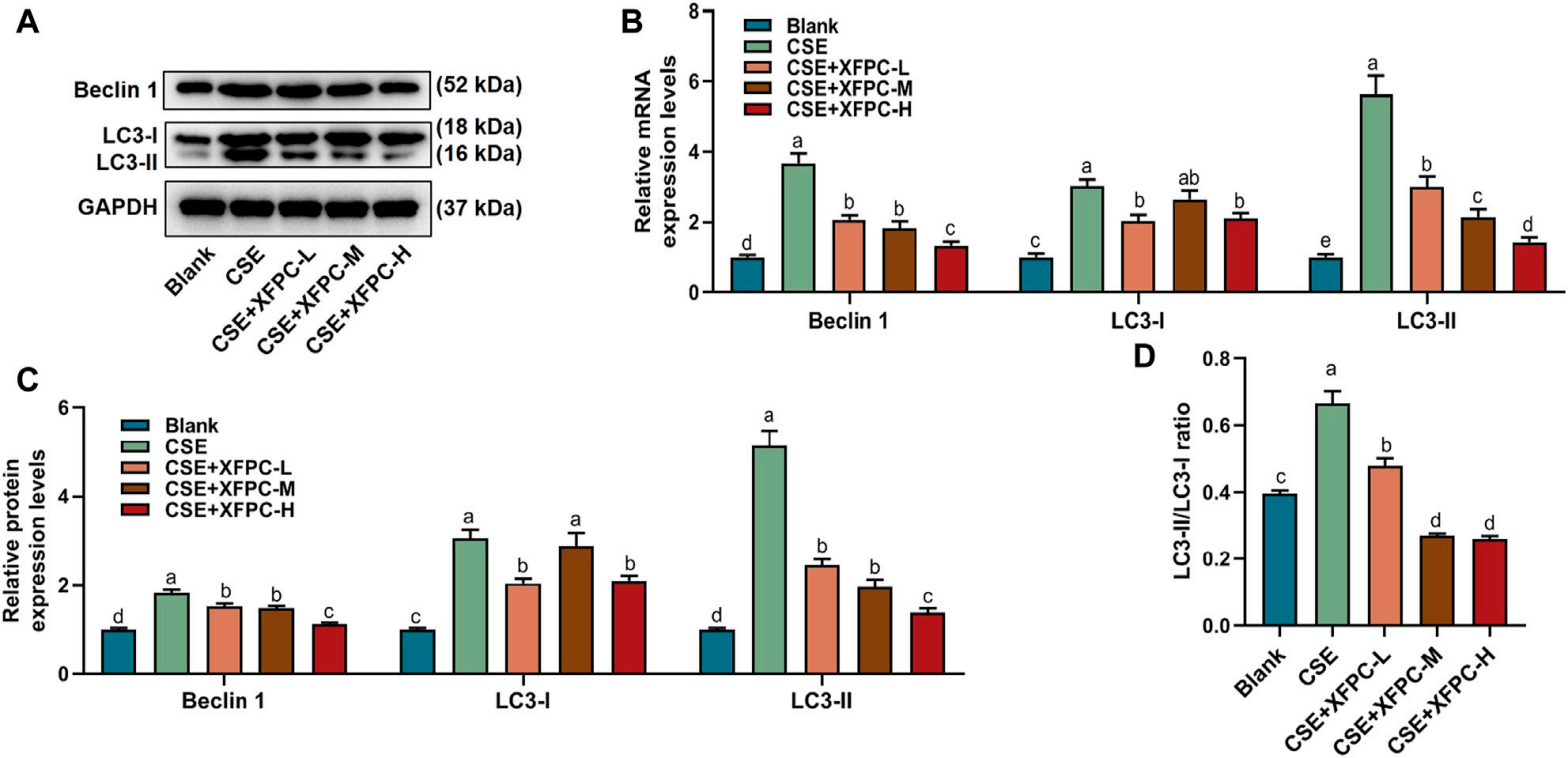
XFPC could inhibit expression of autophagy in CSE-HBE cell model. **(A)** The protein level of autophagy indicators, Beclin 1, LC3-I, and LC3-II were detected by western blot in HBE cells which were treated with CSE and different concentrations of XFPC. **(B)** Quantification of mRNA expression level of Beclin 1, LC3-I and LC3-II. **(C)** Quantification of protein levels of Beclin 1, LC3-I and LC3-II from western blot of panel **(A)**. **(D)** The relative protein expression level of LC3-II compared with LC3-I from western blot of panel **(A)**. XFPC, Xuanfei Pingchuan Capsules; CSE, cigarette smoke extract; HBE cells, human bronchial epithelial cells; LC3, microtubule-associated protein light chain 3.

In the CSE-HBE cell model, the autophagy indicator protein LC3-I, LC3-II, and Beclin 1 were significantly increased, while their expression levels were reduced after treating the cells with different doses of XFPC medicated serum (Figures 3A,C). Especially, the ratio of membrane LC3-II compared with cytoplasm LC3-I, which indicates the level of autophagy, were also rescued by XFPC cured in CSE-induced HBE cells (Figure 3D). The similar expression patterns were also detected by RT-qPCR in mRNA level in CSE and XPFC treated cells (Figure 3B). All experiments above suggested that XFPC inhibited expression of autophagy indicators in CSE-HBE cell model both in mRNA and protein level.

### XFPC Inhibited the Level of p38 Phosphorylation in the CSE-HBE Cell Model

**FIGURE 4:**
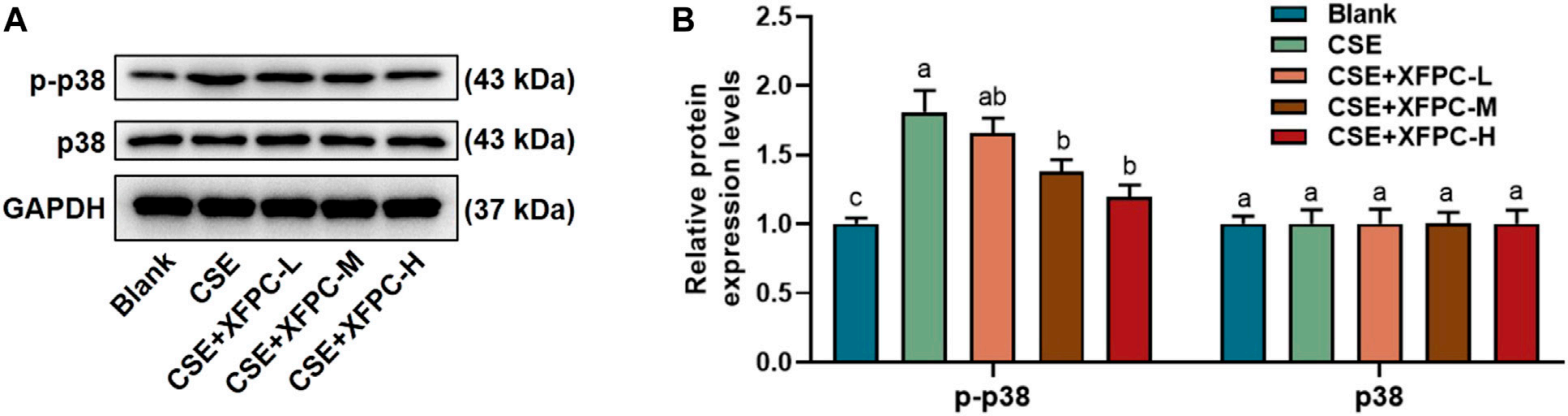
XFPC could inhibit the level of p38 phosphorylation in CSE-HBE cell model. **(A)** The HBE cells were treated with CSE and different concentrations of XFPC. p38 and p-p38 proteins were detected by western bolt analysis. **(B)** Quantification of the levels of p38 and p-p38 in protein level. Lowercase letters indicate statistically significant differences (*p* < 0.05) according to ANOVA followed by Tukey’s HSD test. XFPC, Xuanfei Pingchuan Capsules; CSE, cigarette smoke extract; HBE cells, human bronchial epithelial cells; p-p38, phospho-p38.

After treating HBE cells with CSE, the phosphorylation protein level of p38 was significantly increased. Then cells were treated with different doses of XFPC medicated serum. After XFPC treatment, the p38 phosphorylation protein level was significantly reduced, with the most significant effect seen in the XFPC-H group, while there is no significant change in protein expression of p38 (Figures 4A,B).

### XFPC Regulated Autophagy Levels by Inhibiting p38

**FIGURE 5:**
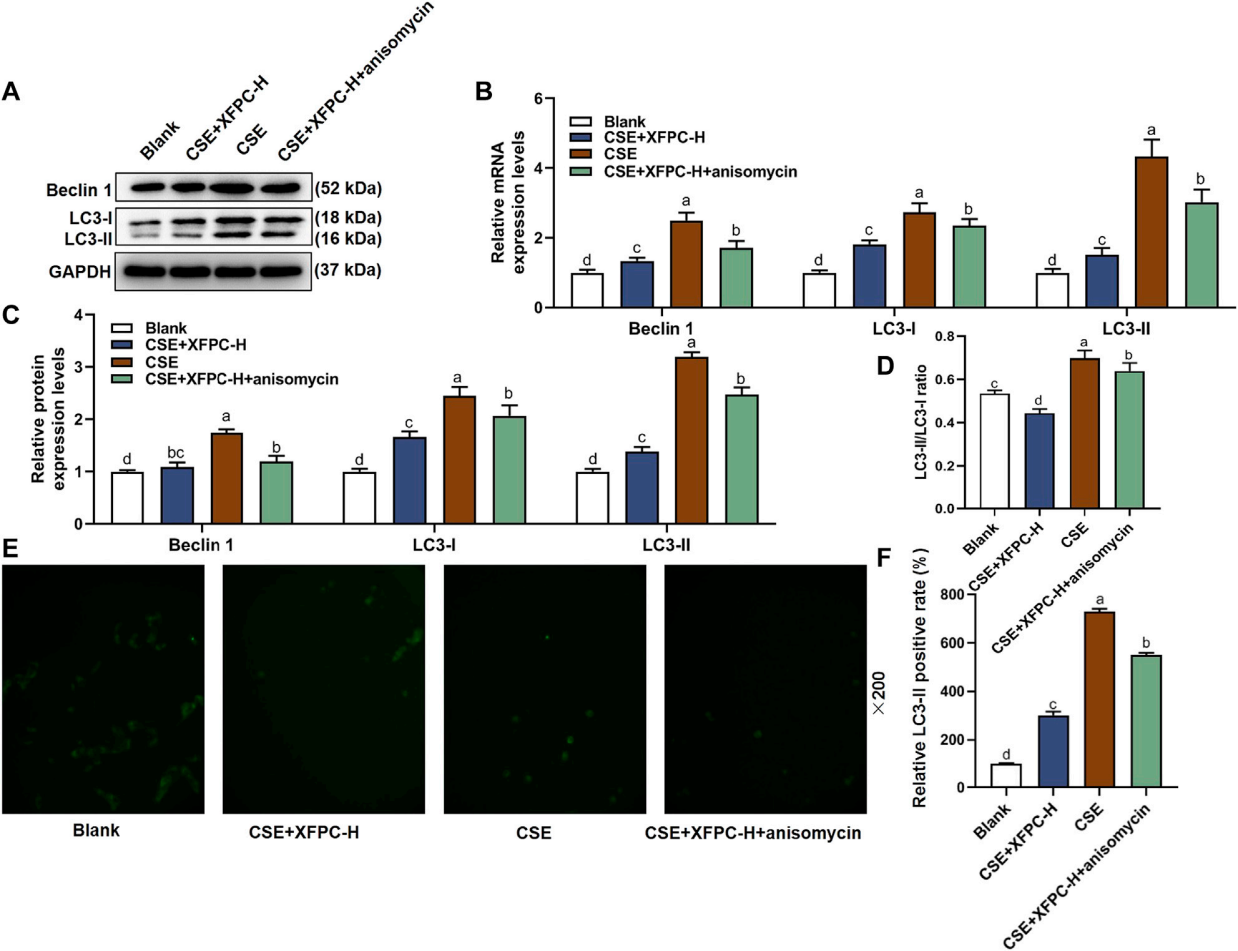
XPFC reduces CSE-induced autophagy in HBE cells by inhibiting JNK pathway. **(A)** Effect of XFPC-H and p38 activator anisomycin on protein expression of autophagy indicator proteins Beclin 1, LC3-I and LC3-II were analyzed by western blot. **(B)** Quantification of the mRNA expression levels of Beclin 1, LC3-I and LC3-II. **(C)** Quantification of protein levels of Beclin 1, LC3-I and LC3-II from western blot of panel **(A)**. **(D)** The relative protein expression level of LC3-II compared with LC3-I from western blot of panel **(A)**. **(E)** Formation of LC3-II puncta in HBE cells was analyzed by immunofluorescence under fluorescence microscopy (×200). **(F)** The relative LC3-II positive rate indicating level of autophagy was shown. Lowercase letters indicate statistically significant differences (*p* < 0.05) according to ANOVA followed by Tukey’s HSD test. XFPC, Xuanfei Pingchuan Capsules; CSE, cigarette smoke extract; HBE cells, human bronchial epithelial cells; LC3, microtubule-associated protein light chain three; p-p38, phospho-p38.

In the CSE-HBE cell model, the autophagy indicator protein LC3-I, LC3-II, and Beclin 1 were significantly increased, while their expression levels were reduced after treating the cells with XFPC-H medicated serum. Following XFPC-H medicated serum treatment of the CSE-HBE cells, we then added the activator of p38, anisomycin, the protein expression of LC3-I, LC3-II, and Beclin 1 increased significantly (Figures 5A,C). Especially, the ratio of membrane LC3-II compared with cytoplasm LC3-I, which indicates the level of autophagy, were also rescued by XFPC-H cured in CSE-induced HBE cells (Figure 5D). The similar expression patterns were also detected by RT-PCR in mRNA level in CSE and XPFC treated cells (Figure 5B). Further observation in immunochemistry method found autophagy in the XFPC-H group could be increased by anisomycin (Figures 5E,F). All experiments above suggested that XFPC-H inhibited expression of autophagy indicators in CSE-HBE cell model both in mRNA and protein level, and the p38 activator can reverse the effect.

### XFPC Promoted Cell Growth by Inhibiting p38 Phosphorylation

**FIGURE 6:**
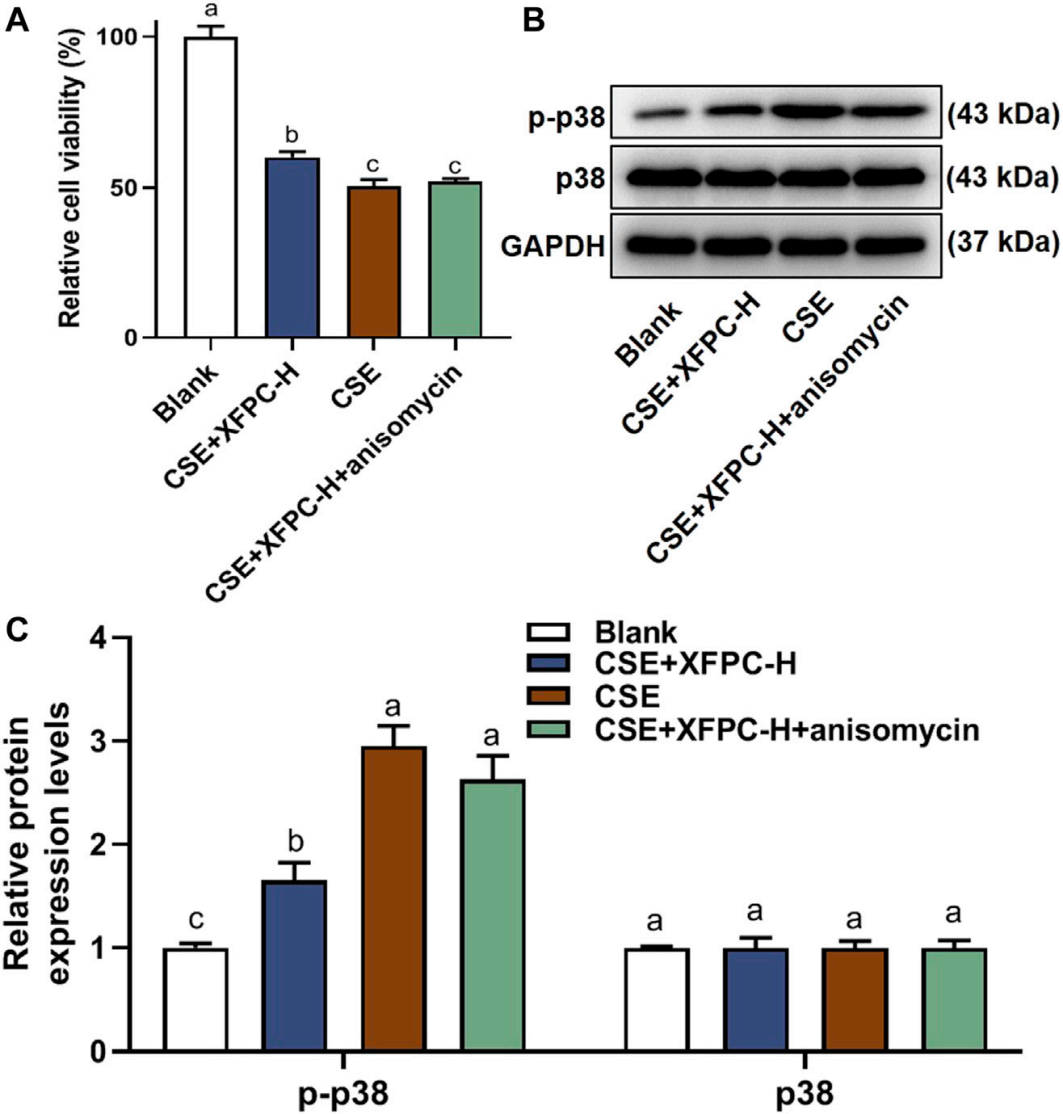
XFPC could promotes cell growth by inhibiting p38 phosphorylation. **(A)** Effect of XFPC-H and p38 activator anisomycin on cell viability in CSE-exposed HBE. Data are presented as mean ± SD from three independent experiments. **(B)** The protein expression of p38 and p-38 in HBE cells after XFPC-H or anisomydin treated. **(C)** Quantification of the levels of p38 and p-p38 in protein level of panel **(B)**. Lowercase letters indicate statistically significant differences (*p* < 0.05) according to ANOVA followed by Tukey’s HSD test. XFPC, Xuanfei Pingchuan Capsules; CSE, cigarette smoke extract; HBE cells, human bronchial epithelial cells.

Compared to the blank group, CSE exposure inhibited HBE cell viability significantly, while the treatment with XFPC-H medicated serum improved cell viability. After adding anisomycin to the XFPC-H group, the improving effect of XFPC on cell viability was partially reversed (Figure 6A). In the CSE-HBE cell model, the protein of p38 phosphorylation was significantly increased, while its expression level was reduced after treating the cells with XFPC-H medicated serum. Following XFPC-H medicated serum treatment of the CSE-HBE cells, we then added the activator of p38 anisomycin. The phosphorylation level of p38 was significantly increased in the XFPC-H cells treated with anisomycin compared to the XFPC-H cells not treated with anisomycin. The treat effect of XFPC on p38 phosphorylation was partially reversed. Meanwhlie, there is no significant change in protein expression of p38 (Figures 6B,C). It suggested that XFPC improved the cells viability and inhibited the phosphorylation level of p38 in CSE-HBE cell model.

## Discussion

COPD is a respiratory disease characterized by progressive airflow limitation and persistent respiratory symptoms. COPD has severely endangered public health and significantly declines patient quality of life (Singh et al., 2019). Autophagy is pathway by which damaged and senescent tissues are degraded in lysosomes (Sosulski et al., 2015). The production of amino acids and other raw materials required for cell metabolism during autophagy is an important method of maintaining cell homeostasis. Recently, many studies have analyzed the relationship between autophagy and COPD (Husebo et al., 2016; Russell et al., 2016; Mizumura et al., 2018).

From the perspective of Traditional Chinese Medicine, COPD pathogenesis is caused by a lack of qi in the lungs and the spleen and kidney inadequacy, which leads to unfavorable qi, sputum obstructing the airway, and symptoms such as tightness and fullness in the chest and shortness of breath. COPD belongs to the category of “lung distension” in Traditional Chinese Medicine. The main treatments are to resolve phlegm and relieve asthma in the onset period, and to invigorate the lungs, spleen, and kidneys during the remission period. XFPC can relieve asthma, reduce phlegm, and alleviate cough. Therefore, their indications are consistent with the main pathogenesis of COPD.

XFPC are composed of ingredients that relieve asthma, phlegm, and cough. Ephedrine and pseudoephedrine, the active ingredients of ephedra in XFPC, have an anti-asthmatic effect by preventing the release of allergic mediators. The effective components of baicalein and baicalin affect the metabolism of arachidonic acid and inhibit the production of prostaglandins and leukotrienes, thereby reducing wall permeability, leukocyte chemotaxis, and the expansion of blood vessels by inflammatory mediators (Peng et al., 2019). Other ingredients, such as cortex mori radices, lepidium seed, perillaseed, and Asters can inhibit the release of the inflammatory mediators tumor necrosis factor α, interleukin 8, and interleukin 1β, and have been referred to as “Chinese medicine antibiotics” (Sohn et al., 2004; Chang et al., 2011). XFPC have been clinically used at Shanxi Provincial Hospital of Traditional Chinese Medicine for more than 10 years, with an average annual clinical use of >50,000 boxes in the hospital. XFPC are effective for COPD patients with symptoms such as cough, sputum, and asthma (Xiaoming et al., 2012; Yang et al., 2016).

Autophagy is a process of targeted programmed death of eukaryotic cells of tissues with dysfunction. Autophagy is important for maintaining cell homeostasis, but excessive autophagy can cause cell dysfunction. Studies have found that the autophagy-related protein Beclin 1 and the apoptosis-related protein Bcl-2 or Bcl-XL bind to each other, causing Beclin 1 to form phagocytic vesicles. Under normal conditions, the phagocytic vesicles extend the enveloping mitochondria under the effect of LC3, mature, and then combine with lysosomes to trigger mitochondrial (Johnson et al., 2005; Cao et al., 2020).

XFPC have a significant effect in the early clinical treatment of COPD, alleviating the symptoms of cough and excess sputum, improving lung function, and reducing the number of acute exacerbations in patients (Xiaoming et al., 2012; Xiaoming et al., 2017). Animal experiments have confirmed that XFPC can reduce the adhesion and shedding of cilia in Sprague Dawley rats, and inhibit the proliferation of goblet cells. Morphology showed reduced focal necrosis of the lung and restored lung elasticity. Light microscopy revealed reduced inflammatory cells in the trachea and blood vessels of the lung tissue, reduced secretions, and reduced pulmonary bullae. Previous reports have shown that XFPC can significantly improve airway inflammation in COPD rats, reduce the changes in airway tissue structure, and reduce the expression of inflammatory factors in the serum (Xiaoming et al., 2012; Xiaoming et al., 2015; Xiaoming et al., 2016; Xiaoming et al., 2017).

Smoking is the most common identifiable risk factor for COPD, and smokers have a higher COPD mortality rate than non-smokers (Kohansal et al., 2009). Ning et al. (2006) studied the comprehensive gene expression profile of Global Initiative for Chronic Obstructive Lung Disease (GOLD)-2 and GOLD-0 smokers. Their results suggested that autophagy-related protein ATG8/microtubule-related protein-1LC3 is a potential molecular target for COPD. Further research showed that this autophagy protein plays a key role in cigarette smoke-induced emphysema (Chen et al., 2010; Chen et al., 2008).

In this study, we treated HBE cells with CSE, established COPD cell models, and treated the cells using different concentrations of XFPC. After treatment with XFPC, the HBE cells had an elongated shape and increased number, indicating that XFPC were able to reverse cell damage and improve the cell surival rate caused by COPD (Figures 1A, 2A). Further, mRNA and protein expression of LC3-I, LC3-II, and Beclin 1 were significantly increased. The immunofluorescence test of LC3-II protein also showed that CSE activated autophagy in HBE cells, and XFPC could reduce the level of autophagy in HBE cells (Figure 2B), indicating that the level of autophagy was increased in the COPD cell model (Figures 3A–C).

p38 is an important member of the mitogen-activated protein kinase family. It participates in the production of various inflammatory cytokines and the conduction of stress signals, and plays an important role in cell differentiation and apoptosis (Tamura et al., 2004). The p38 pathway is significantly related to the regulation of autophagy; thus, inhibition of p38 phosphorylation can significantly reduce the level of autophagy (Li et al., 2016). The cells that activate this pathway inhibit cell proliferation. p38α can force the cell cycle to enter the quiescent phase and promote DNA repair to resist chemotherapy-induced DNA damage (Zarubin et al., 2005).

In this study, we found that XFPC significantly inhibited the phosphorylation level of p38 and the expression of autophagy-related proteins in the CSE-HBE cell model, suggesting that p38 phosphorylation may be a target for XFPC autophagy inhibition. When a p38 activator and XFPC interfered with the CSE-HBE model cells simultaneously, the phosphorylation level of p38 was significantly higher than that of XFPC intervention, Beclin 1, LC3-I, and LC3-II mRNA and protein expression increased, and the immunofluorescence test of LC3-II protein also increased (Figures 5A–F). Cell viability was significantly decreased in the CSE-HBE model. XFPC improved cell viability; however, the p38 agonist anisomycin reversed the beneficial effect of XFPC on cell viability (Figure 6A). This indicates that XFPC promote the viability of CSE-induced HBE cells but that p38 pathway activation inhibits the viability of CSE-induced HBE cells. Therefore, we conclude that XFPC can significantly inhibit CSE-induced autophagy levels in HBE cells. Based on these results, we propose that the p38 phosphorylation level is directly proportional to the COPD cell model. We suggest that XFPC repair the COPD cell model by inhibiting p38 phosphorylation and reducing the level of autophagy.

## Conclusion

Our results show that XFPC significantly inhibits the level of autophagy; decreases the expression of p-p38, Beclin 1, LC3I, and LC3II; reduces cell apoptosis; and increases cell viability in a CSE-induced HBE cell model. However, after adding a p38 agonist, the therapeutic effect of XFPC was weakened. Therefore, XFPC have the potential to ameliorate autophagy by suppressing p38 phosphorylation in CSE-HBE cells.

## Data Availability

The original contributions presented in the study are included in the article/Supplementary Material, further inquiries can be directed to the corresponding author.

## Abbreviations

BSA, bovine serum albumin; COPD, chronic obstructive pulmonary disease; CSE, cigarette smoke extract; CT, cycle threshold; GAPDH, glyceraldehyde 3-phosphate dehydrogenase; GOLD, Global Initiative for Chronic Lung Disease; HBE, human bronchial epithelial cells; LC3, microtubule-associated protein light chain 3; p-p38, phospho-p38; RT-PCR, real-time polymerase chain reaction; SDS, sodium dodecyl sulfate; TBS, tris-buffered saline; XFPC, Xuanfei Pingchuan Capsules; XFPC-L, low XFPC dose; XFPC-M, medium XFPC dose; XFPC-H, high XFPC dose.
